# Social and physical activity as rehabilitation for preschool kids with cancer—study protocol for a randomized controlled trial

**DOI:** 10.3389/fped.2026.1768975

**Published:** 2026-04-13

**Authors:** Anna Pouplier, Martin Kaj Fridh, Peter Schmidt-Andersen, Marianne Hoffmann, Helle Winther, Jan Christensen, Hanne Bækgaard Larsen

**Affiliations:** 1 Department of Paediatrics and Adolescent Medicine, Juliane Marie Centre, Copenhagen University Hospital—Rigshospitalet, Copenhagen, Denmark; 2 Department of Occupational Therapy and Physiotherapy, Copenhagen University Hospital—Rigshospitalet, Copenhagen, Denmark; 3 Department of Nutrition, Exercise and Sports, Faculty of Science, University of Copenhagen, Copenhagen, Denmark; 4 Department of Clinical Medicine, Faculty of Health and Medical Sciences, University of Copenhagen, Copenhagen, Denmark

**Keywords:** cancer, physical activity, preschoolers, protocol, randomized controlled trial, rehabilitation, social activity, structured active play

## Abstract

**Background:**

Preschool children diagnosed with cancer are at risk of delays in motor, cognitive, personal, and social development, all of which are closely connected to being physically active and playing. This study protocol describes the rationale and methods for testing a multimodal intervention in a two-arm, randomized controlled superiority trial. The trial investigates the effect of a 9-month rehabilitation program incorporating physical activity at both the hospital and at home on gross motor function in preschool children diagnosed with cancer.

**Methods:**

The Social and Physical Activity as Rehabilitation for preschool Kids with cancer (SPARK) intervention includes the following components: 1) Supervised group-based social and physical activity at the hospital; 2) A parent educational program, and 3) Online supervised group-based social and physical activity in the families’ homes. The group-based social and physical activity is conducted as structured active play. We will include 82 preschool children (aged 1–5 years), newly diagnosed with cancer, who will be randomly assigned on a 1:1 ratio to either group (a) or group (b). Group (a) will receive supervised group-based social and physical activity at the hospital (component 1), and group (b) will receive supervised group-based social and physical activity at the hospital, the parent education program, and online supervised group-based social and physical activity at home (component 1–3). The primary outcome is gross motor function measured using the Peabody Developmental Motor Scales, Third Edition (PDMS-3). Secondary outcomes are parents’ knowledge of physical activity and the children's general physical function. Assessments will be conducted at treatment initiation (baseline), and 6, 9 (primary endpoint), and 12 (follow-up) months after treatment initiation. Additionally, we will qualitatively explore the parents’ and children's experiences, the rehabilitation program's potential for the children's social development, and the importance of professionals in facilitating structured social and physical activities.

**Discussion:**

We expect this study protocol to enhance clarity and transparency while providing insights for clinicians and researchers interested in the gross motor development of preschool children with cancer during treatment. By combining hospital-based and home-based social and physical activity with parental education, this trial has the potential to transform rehabilitation in preschoolers during cancer treatment.

## 1 Introduction

Participating in physical activity and active play (defined as unstructured and structured activities including any bodily movements (1, 2)) is crucial for healthy growth and development in early childhood, positively impacting motor, cognitive, personal, and social development (3–5). However, preschool children diagnosed with cancer are at risk of developmental delays and impairments in these areas due to the treatment-related side effects and sedentary behavior (6). Studies have shown that these children often experience substantial challenges with fine and gross motor skills, including severe gait difficulties (6), which limit opportunities for physical exploration and progression of motor skills during a critical developmental period. Being absent from their usual social environments, such as daycare, limits their social interactions with peers and the development of social skills (7). When the children return to their regular everyday life, they often struggle to keep up physically with their peers, lack confidence in their movement abilities, and feel socially excluded (8). As early childhood is a formative developmental period, interruptions caused by cancer treatment make it essential to develop rehabilitation approaches to support their social and physical development during and after treatment.

The previous—“Rehabilitation including structured active Play for preschoolers with cancer” (RePlay) trial (9)—originating from the same research environment and study population as the present work, offering preschoolers with cancer systematic, supervised physical activity as structured active play—demonstrated successful recruitment and high relevance for families (10). The children expressed joy of movement and a desire to play, and the intervention supported them in regaining confidence in their movement and in practicing social skills (11). The parents expressed how the systematic approach and structured intervention were essential in supporting their child to be physically active (12). However, logistical challenges and the need for more home-based physical activity support were identified as areas for improvement (10, 12).

Building on the RePlay trial's findings, the new Social and Physical Activity as Rehabilitation for preschool Kids with cancer (SPARK) intervention aims to address these issues by offering a more flexible and accessible multimodal intervention.

### 1.1 Study aims

The SPARK trial aims to investigate the effect of a nine-month multimodal intervention consisting of supervised group-structured social and physical activity at the hospital, combined with a parent educational program, and online supervised group-based social and physical activity at home, on preschoolers with cancer's gross motor function development compared to solely supervised group-based social and physical activity at the hospital. Subsequently, we aim to investigate the effects of the intervention on children's functional skills in everyday activities and on parents’ knowledge of physical activity. Furthermore, during the study period, we aim to qualitatively explore the impact of the intervention.

## 2 Methods and analyses

This protocol is reported in accordance with the SPIRIT 2025 updated guideline for reporting protocols of randomized trials (13).

### 2.1 Study design

**Figure 1:**
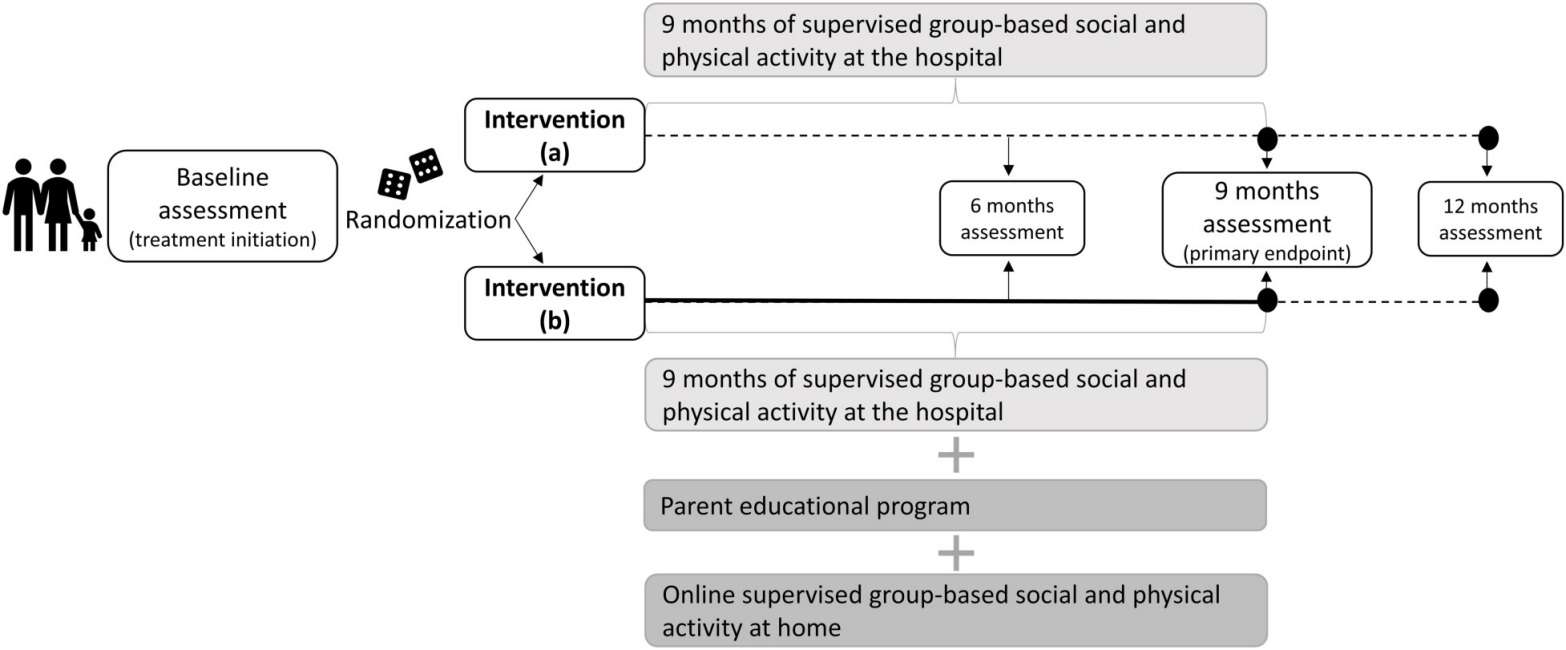
Study design of the SPARK randomized controlled superiority trial.

The study is a single-center randomized controlled superiority trial comparing two intervention arms, as illustrated in Figure 1. The primary endpoint of the study will be at the end of intervention at nine months post-treatment initiation, with follow-up at 12 months post-treatment initiation. Quantitative and qualitative data will be collected at defined intervals during the study and reported separately for dissemination. The study is registered at ClinicalTrials.gov: NCT07213024.

### 2.2 Setting

Participants will be recruited from the Department of Paediatrics and Adolescent Medicine at Copenhagen University Hospital—Rigshospitalet, a public hospital in Denmark financed via up-front tax payment. The department provides treatment for approximately 50% of all newly diagnosed children with cancer in Denmark.

### 2.3 Eligibility criteria

Children eligible to participate in the study must be: (1) newly diagnosed with cancer or cancer-like benign disorders; (2) between the ages of 1–5 years old (i.e., ≥12 to <72 months) at the time of treatment initiation; (3) undergoing intravenous chemotherapy and/or radiation therapy and/or immunotherapy at the Department of Paediatrics and Adolescent Medicine at Copenhagen University Hospital—Rigshospitalet. Children considered ineligible are those: (1) who are diagnosed with severe mental or physical disorders that prevent them from following instructions in relation to the intervention and testing; (2) for whom neither of the parents/guardians can communicate in Danish without a translator.

### 2.4 Recruitment

Eligible participants are recruited through the treating physician in the Department of Paediatrics and Adolescent Medicine, who provides the families with information about the trial. If the families are interested, they receive detailed verbal and written information about the trial from a trial representative within 14 days of treatment initiation. The parents are given a reasonable time (i.e., at least 24 h, usually 24–48 h) to consider participation, after which a trial representative follows up in person or over the phone. If they are willing to participate, written informed consent for participation will be obtained before any study-related activities are conducted.

### 2.5 Randomization

Following the baseline assessment, participants are randomly allocated to either intervention group (a) or intervention group (b), using centrally administered, computer-generated (REDCap) random numbers and a secure, concealed allocation procedure. The participants will be randomized in blocks with varying block sizes, which are unknown to the research team. The block size is chosen by an external researcher unaffiliated with the trial. The trial representatives, who include the children, are blinded to the randomization strategy (i.e., block size and allocation sequence). The trial representative collects demographic data upon inclusion, and after completion of the baseline assessments, the data are registered in REDCap, which generates the random group allocation. Randomization is stratified by age at inclusion (respectively, <36 and ≥36 months old) and by diagnostic group (hematologic cancers, central nervous system tumors, and extracranial solid tumors). The stratification by age is determined at 36 months to respect age-specific development and is in line with age-group divisions outlined in several guidelines (2, 14). Due to the nature of the intervention, neither the children nor the parents are blinded to their group allocation.

### 2.6 Intervention

The SPARK trial consists of two intervention groups. Both groups will receive usual care which include occupational therapy and physiotherapy if needed. Intervention group (a) includes nine months of supervised group-based social and physical activity at the hospital conducted as structured active play sessions. Intervention group (b) comprises a nine-month program of supervised group-based social and physical activity at the hospital, conducted as structured active play sessions, combined with a parent educational program, and supervised group-based social and physical activity delivered online for implementation at home. The different intervention components are described in detail below.

#### 2.6.1 Supervised group-based social and physical activity at the hospital

The hospital-based part of the intervention includes daily supervised group-based social and physical activity conducted as structured active play sessions during the weekdays (i.e., Monday–Friday) at the hospital for nine months. All sessions are planned as 45 min sessions with different playful movement activities, structured using the RePlay Model (15) following four core principles: 1) ritual practices; 2) reinforcement of movement through repetition; 3) development through appropriate challenges; and 4) adjusting activities to accommodate treatment-related side effects. The structure entails a repeated starting ritual followed by a familiar activity that all children can master to induce familiarity and success. The main part of the structured active play session consists of active play activities that aim to motorically challenge the children within their zone of proximal development (15, 16). As a consequence of the medical treatment, the children's physical capabilities fluctuate from day to day, and accordingly, the activities will be adjusted on an individual level to accommodate these fluctuations. All sessions conclude with a familiar activity that the children can master, followed by a repeated closing ritual. All activities are conducted in groups, with healthcare professionals, parents, and children participating together to support social development. The group-based active play sessions will take place at the pediatric oncology ward and will be supervised by a health professional (i.e., an exercise professional or physiotherapist). Respecting age-specific development, children will be divided into two groups: <36 months and ≥36 months. Admitted families who are prevented from participation in the group sessions, either due to isolation, procedures or health-related conditions, are offered an individual session in their hospital room, supervised by a healthcare professional. A child can switch from group sessions to individual sessions during a hospital stay, the individual sessions are offered to ensure a possibility for participation.

#### 2.6.2 Parent educational program

The parent educational program consists of two 30–60 min educational sessions within the first two months of the child's treatment trajectory. The sessions aim to provide parents with the knowledge and skills to facilitate structured, active play at home. Session 1 provides general information on gross motor function development, common physical side effects of cancer treatment, and the importance of physical activity. Session 2 includes an interactive introduction to structured active play and the RePlay Model, including the inspiration for active play activities that can be done at home to challenge different gross motor skills. The parents will receive practical materials on gross motor development stages and skills, as well as descriptions and access to videos of active play activities that target gross motor functions. The parents are encouraged to facilitate and partake in structured active play with their child daily when they are not at the hospital, and to participate in the online supervised group sessions.

#### 2.6.3 Online supervised group-based social and physical activity at home

To support the parents in facilitating structured active play at home, we will provide online supervised group-based social and physical activity at home. These sessions will be available daily during weekdays (i.e., Monday–Friday). Here, the families can log on and have a healthcare professional (i.e., an exercise professional or physiotherapist) guide them through active play activities. The online supervised session requires parents to be the main facilitators in their home, guided by a healthcare professional who will initiate the session with a ritual and a familiar activity, as described above, and conclude it with a ritual. To accommodate families who are unable to join the online supervised sessions due to, for example, logistical constraints, they will have access to pre-recorded sessions they can follow at home at a time convenient for them. The online supervised group-based sessions serve as a supporting element for parents, providing them with an opportunity to systemize structured active play at home.

Each family will receive basic essential home-based active play equipment (e.g., bean bags, balls, bingo games, items for an obstacle course) for the online sessions. The sessions will be conducted through video connections (Teams or Skype).

### 2.7 Outcomes

Assessment of outcomes for both groups will be conducted within 14 days of treatment initiation (t0—baseline), at 6 months after treatment initiation (t1), 9 months after treatment initiation (t2—primary endpoint), and 12 months after treatment initiation (follow-up) (t3). The baseline, 6-, and 12-month assessments are aligned with the RePlay trial's assessment timepoints (9), enabling cohort comparison. An overview of the study design, including enrollment and assessments, is summarized in Table 1.

**Table 1:** Schedule of enrollment, interventions, assessments of participants, and additional data collection in the SPARK trial.

	STUDY PERIOD					
	Enrolment	Pre-allocation	Post-allocation			
			Intervention			Follow-up
**TIMEPOINTS**	TTI	**T0** (**Baseline**)	T0^1^	**T1**	**T2** (**primary end-point**)	**T3**
**ENROLMENT:**						
Eligibility screening	X					
Oral and written information	X					
Informed consent		X				
**ASSESSMENTS:**						
PDMS-3		X		X	X	X
PEDI		X		X	X	X
Parent knowledge test on physical activity and gross motor development		X	Pre and post the parent educational program^b^		X	X
**INTERVENTION:**						
Usual care^a^ (i.e., occupational- and physiotherapy if needed)	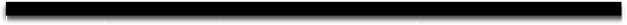					
Supervised active play at the hospital^a^			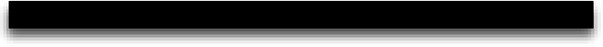			
Online supervised active play at home^b^			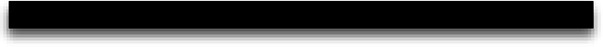			
Parent educational program^b^			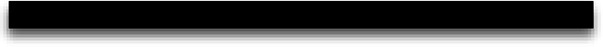			
**ADDITIONAL DATA:**						
Medical record info (sex, age, diagnosis, date of diagnosis, date of treatment initiation, date of relapse, possible date of death, comorbidity, treatment, and medical care)	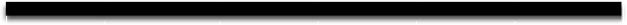					
Socio-demographics		X				
Adverse events			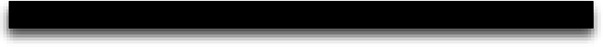			
Feasibility assessment			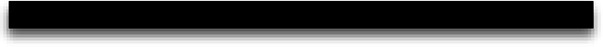			
**QUALITATIVE EXPLORATION:**						
Interview			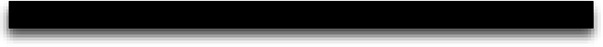			
Informal conversations			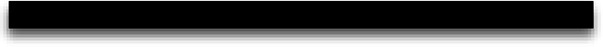			
Observation			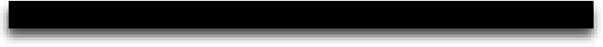			

TTI, treatment initiation, T0 =  + 1–14 days after TTI (Baseline), T0^1^ = +1–14 days after TTI, T1 = 6 months (±14 days) after TTI, T2 = 9 months (±14 days) after TTI (post-intervention, primary end-point), T3 = 12 months (±14 days) after TTI.

^a^ Intervention group (a) and (b).

^b^ Only intervention group (b).

#### 2.7.1 Primary outcome measure

##### 2.7.1.1 Gross motor function

To measure gross motor function, we use the gross motor function composite of the Peabody Developmental Motor Scales, Third Edition (PDMS-3), which assesses total gross motor function in children aged 0–6 years. To assess overall gross motor function, we use the sum of the scaled scores for the three gross motor subtest: body control (56 items), body transport (63 items), and object control (39 items) (17). All items within the subtests are scored individually with a score of 0 (i.e., the child cannot or will not attempt the item, or the attempt does not show that the skill is emerging), 1 (i.e., the child's performance shows a clear resemblance to the item mastery criteria but does not fully meet the criteria), or 2 (i.e., the child performs the item according to the criteria specified for mastery) (17). The raw score for each subtest is the sum of all the item scores. The raw scores are converted to scaled scores for each subtest, which account for age differences, and the subtest scaled scores are summed to an overall scaled score. We use the summed gross motor scaled scores for comparison between the groups. The summed scaled score can also be converted to an index score of the gross motor composite.

The assessment is administered by two trained members of the project team. The test is conducted by starting with a given item in the subtest, depending on the child's age (specified in months). The items are treated singularly until the child receives a score of 2 on three consecutive items, determining the lower level. It may be necessary to revert to earlier items to achieve three consecutive scores of 2. The preceding items are given a score of 2. The test then proceeds with further items until the child receives a score of 0 on three consecutive items, determining the ceiling level. The remaining items are then given a score of 0. The test has shown acceptable psychometric properties, including Cronbach *α* = 0.88–0.96, test–retest *r* = 0.94–0.98 (17). The previous Peabody Developmental Motor Scales, Second Edition (PDMS-2) gross motor function composite (previously called subtest) has been shown to be feasible to use for preschoolers with cancer during treatment, with 80% of the children completing the full composite at 6 months post-treatment initiation (10).

#### 2.7.2 Secondary outcome measures

##### 2.7.2.1 Level of everyday function

To measure the children's level of everyday function, we use the Pediatric Evaluation of Disability Inventory (PEDI), which assesses the capability and performance of selected functional activities of children aged 6 months to 7.5 years old through three different scales: functional skills, caregiver assistance, and modifications (18, 46). In this study, the modification scale is not used. The functional skills scale is divided into three domains: self-care (73 items), mobility (59 items), and social function (65 items), with each item scored as 0 or 1. The caregiver assistance scale is divided into three domains: self-care (8 items), mobility (7 items), and social function (5 items), with each item scored 0–5. Aggregate scores are defined as the sum of each domain and are converted to normative standard scores for each domain (18). PEDI is assessed by a trained member of the project team and is conducted as a structured interview with parents. The PEDI assessment has been shown to be feasible to use with parents of preschoolers with cancer during treatment, with a 95% completion rate at 6 months post-treatment initiation (10).

##### 2.7.2.2 Parent knowledge test on physical activity and gross motor development

We developed a Multiple-Choice Assessment with a one-best-answer (scored correct/incorrect) format to evaluate the parent educational program by testing parents’ knowledge of physical activity, gross motor development, and structured active play. The development followed design and development recommendations (19–21).

#### 2.7.3 Qualitative explorations

The qualitative explorations are rooted in practitioner research and Van Manen's hermeneutic-phenomenological approach, where the purpose is to describe and understand the children's and parents’ experiences with the intervention (22, 23). We have included appropriate qualitative explorations that will be conducted concurrently with the randomized controlled trial and reported separately.

##### 2.7.3.1 Semi-structured interview

We will conduct semi-structured interviews with parents to elicit their lived experiences with both the supervised in-hospital and at-home group-based sessions (24) and to explore how they experience the intervention in regards to their child's physical, social and personal development. By purposeful criterion sampling, we will gather a broad perspective where parents of children with diverse characteristics (e.g., age, diagnosis, and participation rates in intervention sessions) will be interviewed (25). The interview will be conducted by an experienced member of the research group and will preferably be done face-to-face. The interviews will be carried out while the families are actively participating in the intervention (i.e., approximately halfway through the intervention at 4 months) to elicit their lived experiences.

##### 2.7.3.2 Observations and informal conversations

To ensure the children's voices, we will conduct close participant observations during the supervised in-hospital and at-home group-based sessions. Rooted in a hermeneutic-phenomenological approach, these methods are chosen with the purpose of describing and understanding the children's lived experiences (23). The observations will be conducted throughout the first year of the intervention using the observation method of “observing participants” (26). The professionals who facilitate the group-based sessions (a maximum of two professionals are present in a session) are observant of the children's verbal and bodily expressions, interactions, and the events that occur during the sessions (23, 26). This approach offers a way to explore how children articulate their experiences through their modes of participation (23, 27). After each session, field notes are written as narrative scenic descriptions (11, 28). The observations can also include notes from informal conversations, which can be viewed as unstructured interviews that do not require scheduling and occur in everyday settings. This creates a more natural form of communication, suitable where a traditional interview is not applicable (24, 29).

Additionally, we will conduct partial participant observations (26) to explore the role of the facilitating healthcare professional during the supervised in-hospital and at-home group-based sessions. During these observations, there will be one facilitating professional and one observer who is not part of the facilitating team.

#### 2.7.4 Additional data collection

##### 2.7.4.1 Socio-economic position

Upon inclusion, socio-demographic information from the children and parents is collected through a questionnaire, including place of birth, place of residence, family structure, parent admitted with the child, support from friends/relatives, daycare/preschool attendance, physical and leisure activities of the child and parents, and educational level of the parents.

##### 2.7.4.2 Disease and treatment

The following data are extracted from the children's medical records: sex, age, diagnosis, date of diagnosis, date of treatment initiation, date of relapse, possible date of death, comorbidity, treatment (e.g., treatment protocol, medical treatment), and medical care.

##### 2.7.4.3 Feasibility

The feasibility of the overall intervention and each intervention component will be assessed by acceptability, attrition, adherence, and completion (10, 30). Mild, moderate, and severe adverse events are registered (31). The parents will answer an evaluation survey after the intervention. The evaluation survey will include ratings of the relevance of the different intervention components, the time of day for both the in-hospital and at-home group-based sessions, the facilities for the in-hospital group-based sessions, whether their child was challenged during the sessions, the inspirational material, and the extent to which they used this material. Furthermore, there will be space for the parents to elaborate on their ratings.

### 2.8 Sample size

The following sample size calculation is based on preliminary baseline data from the RePlay study (9) of the sum of standard scores from three gross motor domains (i.e., stationary, locomotion, and object manipulation) of the PDMS-2. With a mean of 20.03 in summed gross motor standard score and a standard deviation of 7.4 (unpublished data from RePlay, *n* = 36) (9), a 25% difference between groups, an alpha level of 0.05, and a power of 80%, we need to include 68 preschool children with cancer. Based on RePlay, we assume 20% missing data of the primary outcome at the primary endpoint (10). Therefore, we aim to include 82 preschool children with cancer. Based on the deficiencies observed within two weeks of treatment initiation (baseline score of 20.03 gross motor function), we expect a 25% difference between groups to be obtainable over 9 months of intervention.

### 2.9 Statistical analysis

The primary outcome [gross motor sum of scaled scores from the three subtests (i.e., body control, body transport, and object control) of the PDMS-3] will be analyzed using analysis of covariance (ANCOVA) models with the residual variance depending on the group [intervention group (a) and intervention group (b)]. To investigate whether the impact of adjusting for the differences between the two groups could result from differences in age, diagnosis, and time since diagnosis, the groups will be compared in three different models: 1) a raw model without any adjustments, 2) a model further adjusted for diagnosis, and 3) time since diagnosis. The secondary outcome of the PEDI will be analyzed using a similar model. The Multiple-Choice Assessment will be analyzed using a Linear Mixed model to investigate post-intervention knowledge between parents in intervention group (a) and intervention group (b) while adjusting for baseline scores. Secondary analyses include within-group changes pre- and post-intervention, analyzed using paired *t*-tests. An alpha level of 0.05 will be considered significant. Descriptive statistics will be used to summarize feasibility outcomes (i.e., acceptance, attrition, completion, adherence, adverse events, and the parent evaluation survey).

### 2.10 Qualitative analysis and rigor

The verbatim interview transcripts, field notes from observations, and field notes from informal conversations, will be analyzed separately using inductive thematic analysis, through the following steps: 1) a holistic approach where transcripts are read several times to obtain an overall understanding of the content; 2) a selective approach where meaningful units are identified; 3) a detailed approach where meaningful units are coded; and 4) meaningful units and codes are summarized into essential themes (32, 33). The credibility of the qualitative findings will be ensured through prolonged engagement in the field, an active search for “negative cases”, and investigator triangulation, with more than one researcher conducting the interviews, observations, and analyses (34). Transferability is ensured by including observations and interviews from many participants, thereby broadening perspectives and experiences. Thick descriptions will be included with direct text passages from observations and direct quotes from interviews, allowing the reader to judge the fittingness of the findings (34, 35).

### 2.11 Data management

All quantitative data are collected in paper form and then manually entered in a secure electronic database (REDCap) by a member of the project team. Paper forms are stored in a locked cabinet at the trial site. As this process can produce data management errors, the data will be cross-checked independently by two members of the project team to ensure its quality. Qualitative data as interview files and observational field notes will be stored in a secure electronic folder and original interview files will be deleted from the voice recorder after transfer.

## 3 Discussion

This protocol reflects the choices made when designing the SPARK trial. It considers the expected challenges, strengths, and dilemmas of conducting a hospital- and home-based intervention for preschool children undergoing intensive cancer treatment, with parents as an important stakeholder, and ensuring viable outcome measures.

### 3.1 The intervention design

Our results from the previous RePlay trial revealed considerable variability in adherence (0%–95%), which was attributed to logistical challenges, flexibility, and the availability of facilities (10). To address these issues in the SPARK trial, we propose a more flexible intervention design that offers the intervention daily, at the ward, near the patients. This approach is expected to enhance adherence by making the intervention more accessible and convenient, as seen in previous physical activity intervention trials for children with cancer (36, 37). Furthermore, preschoolers should be physically active or participate in active play as much as possible every day, with minimal sedentary activity (2); thus, offering the intervention daily will increase their opportunities to be physically active at the hospital. However, there is a shift towards more home-based treatment to reduce hospital stays and the interruption of family routines and normalcy (38, 39). This calls for effective solutions to support parents in physical activity with their child at home. The SPARK trial is designed with an intervention group that includes additional components of a parent educational program and online supervised group-based social and physical activity at home. The online sessions provide children and parents with regular opportunities for physical activity at home. While online physical activity has proven effective for older children (age 4–18 years) with cancer (40), engaging younger children through a screen may be challenging. Therefore, the role of parents is crucial in these sessions, which are designed to be more interactive and guided rather than instructor-led activities. Additionally, the parent educational program is based on the premise that parents who are more knowledgeable about physical activity tend to have more physically active children (41, 42). Still, developing such a program requires careful consideration. Previous research indicates that parents often feel overwhelmed by the numerous treatment-related responsibilities and tasks they must manage at home (7, 43). Results from the RePlay trial also highlighted that parents appreciated having some of the responsibility taken off their shoulders and valued the shared responsibility of their child's physical activity (12). Conversely, parents also expressed uncertainty about how to engage their children in physical activity at home, and they found the structured active play sessions at the hospital to be a valuable source of inspiration (12). In developing the parent educational program, we made deliberate decisions regarding the timing and length of the educational sessions and the amount of information provided to ensure it was manageable for the parents. We want to begin the intervention as early as possible, as research has shown that physical side-effects are already present within the first month of treatment (44), which is why we include the families within the first 14 days of treatment. However, the timing of the parent educational program is planned for the first two months of treatment to accommodate the treatment-related information load that parents will experience during the first month. As part of the normal interactions with the families throughout the intervention, parents may ask for additional information or have questions. Interacting and answering parents’ additional questions is an unavoidable part of the intervention, but it is not structured or approached systematically, and we do not offer additional educational sessions. Additionally, to ensure a feasible design, both the parent educational program and the online supervised structured active play were developed with input from parents of preschool children in active treatment and tested before the onset of the randomized controlled trial.

When deciding on outcome measures in the SPARK trial, we chose to limit the included outcomes to the PDMS-3 in the assessment of the children. This decision was based on feasibility results from the RePlay trial, showing that many factors influenced the feasibility of outcome assessment in the children throughout the treatment trajectory, including health-related issues, pain, and unwillingness (10). Additionally, the length of the outcome assessment, with the inclusion of more outcomes, some of which were not suitable for the age group, contributed to the complexity (10). Simplifying the outcome measures to focus on the most essential aspect of gross motor function may help improve completion rates.

### 3.2 Strengths and limitations

The variability of the intervention can be viewed as both a strength and a limitation. Due to the side-effects of medical treatment—particularly chemotherapy and corticosteroids—the children's physical capacities fluctuate substantially from day to day. Consequently, the activities must be continuously tailored to each child's current condition to align the daily capabilities with the intended motor challenge level. As a result, the actual exposure to the intervention will vary between participants in terms of duration, content, and intensity, and therefore lacks complete uniformity. However, this variability reflects an unavoidable premise of exercising during childhood cancer treatment. Importantly, the intervention is structured within a well-defined and previously tested framework (the RePlay Model) (15), developed and evaluated in the same study population. This ensures the highest possible degree of standardization while maintaining flexibility for individual adjustments. To evaluate the feasibility and quality of the intervention delivery, we will assess adherence and conduct semi-structured interviews.

A challenge in the present study is the heterogeneity of the participant group, as we include all diagnoses. This increases the complexity of data analysis and may limit the generalizability of the results, particularly when evaluating the overall effect of the intervention across diverse clinical profiles with variations in symptoms and treatment protocols. To address this, we have implemented stratification in the randomization process across diagnostic groups. In contrast, including children with all diagnoses provides a broader insight into the entire patient population and enables future subgroup analyses that may inform more targeted approaches. From an ethical perspective, this is a strength because it ensures that every child has access to the intervention, upholding the principle of equity in healthcare by removing barriers to participation.

The study design includes two active intervention groups, which can be regarded as both a strength and a limitation. Because both study arms receive active interventions, the contrast between groups may be reduced. This overlap in intervention components or mechanisms of action can attenuate observed between-group differences and may lead to an underestimation of the true intervention effect. We expect the additional intervention components to be essential, particularly given that families are spending more time at home and that the intervention is tailored to this context. On the contrary, these components have not been previously tested or evaluated in a trial, and despite a small test period prior to the SPARK RCT, their feasibility remains unknown. Still, we expect the anticipated increase in gross motor function of 25% to be attainable as this responds to an increase in standard score by five “points” across all three subtests. Additionally, the design ensures that the social element of the intervention is supported, as all children in the ward can participate in the play activities at the hospital.

As the literature and evidence are still limited on physical activity interventions and initiatives specifically for preschoolers, we chose to include numerous feasibility and evaluation measures and qualitative explorations to ensure that future interventions and initiatives can be tailored to preschoolers’ and their parents’ needs. Qualitative research within a randomized controlled trial can be a strength as it provides a comprehensive understanding of the intervention from the user's perspective (45). This broadens our understanding and allows us to explore other impacts and new questions that arise.

This protocol outlines a clinical trial designed to generate data on the effect of a multicomponent intervention comprising in-hospital supervised group-based social and physical activity, a parent educational program, and at-home online supervised group-based social and physical activity compared with a single-component, involving only in-hospital supervised group-based social and physical activity In addition to evaluating outcomes, the trial will explore the perspectives and experiences of both children and their parents. The results from the SPARK trial are expected to provide valuable evidence for the emerging yet under-researched field of rehabilitation in preschool-aged children undergoing cancer treatment, thereby advancing the development of best practices in this area—potentially transforming rehabilitation in preschoolers during cancer treatment.

## 4 Ethics and dissemination

The SPARK trial adheres to the ethical research principles outlined in the Declaration of Helsinki. The study has been peer-reviewed and approved by the National Committee on Health Ethics Research through the Regional Research Ethics Committee in the Capital Region of Denmark (jr.nr.: H-25047770, and data handling is approved by the Danish Data Protection Agency (jr. nr.: p-2024-17472). Written informed consent is obtained from parents prior to their inclusion in the trial. Parents are informed that participation is voluntary and that they may withdraw their consent at any time, with no consequences for their child's further treatment. All personal or descriptive data is pseudonymized and will be presented in clusters. The interviews and observational field notes are pseudonymized.

### 4.1 Safety and adverse events

Our previous findings from the Rehabilitation including structured active play for preschoolers with cancer (RePlay) trial show that physical activity, such as structured active play and physical assessment, was found safe for preschool children with cancer, regardless of diagnosis (10). Nevertheless, all adverse events, defined as unintended negative consequences experienced during the structured active play sessions and gross motor function assessments are documented, despite the possibility of a causal relationship between the intervention and usual care (31). In relation to daily inclusion safety criteria for physical activity, data on hemoglobin level, platelet and leukocyte counts, temperature, and signs and symptoms of infection are obtained from the treating physician and nurse. As the trial is perceived as a low-risk trial, there is no data monitoring committee, and the principal investigator is responsible for monitoring the safety of the trial.

### 4.2 Dissemination policy

Future results will be presented in peer-reviewed scientific journals and at international conferences. Authorship eligibility will follow the Vancouver Recommendations for authorship.

## Data Availability

The original contributions presented in the study are included in the article/Supplementary Material, further inquiries can be directed to the corresponding author.
